# Genetic Diversity and Putative Origin of the Bait Polychaete, *Diopatra aciculata* Knox and Cameron 1971 (Annelida: Onuphidae), Occurring in South Africa

**DOI:** 10.1002/ece3.74520

**Published:** 2026-11-06

**Authors:** Stephanie Schoeman, Conrad A. Matthee, Kelvin Hull, Carol A. Simon

**Affiliations:** ^1^ Department of Botany and Zoology Stellenbosch University Matieland South Africa; ^2^ Department of Biochemistry, Genetics and Microbiology University of Pretoria Pretoria South Africa

**Keywords:** bait polychaete, invasive species, marine annelid, mt*DNA*, SNPs

## Abstract

The bait polychaete *Diopatra aciculata* is commercially farmed in Australia and is collected as bait in several South African estuaries. Despite its widespread use, the species' geographic origin remains uncertain, hindering management efforts within sensitive estuarine ecosystems. Mitochondrial Cytochrome Oxidase subunit I (mt*COI*) data and ddRAD‐generated single‐nucleotide polymorphisms (SNPs) were used to gain insight into the origin and genetic diversity of *D. aciculata* . A total of 204 individuals were collected from an aquaculture farm in Australia and the Knysna, Keurbooms, and Swartkops Estuaries in South Africa. The mt*COI* analyses supported the monophyly of *D. aciculata* and revealed lower genetic diversity in the South African population compared to the Australian population. Both marker sets revealed significant genetic differentiation between the continental populations that are naturally isolated by the Indian Ocean basin. The SNP dataset revealed low intrapopulation genetic diversity for both the Australian and South African populations. The discordant genetic diversity findings between the mt*COI* and SNP datasets may be attributed to the differences in the evolutionary rate of the two marker systems. In addition, the low genomic diversity observed in the SNP dataset is consistent with the effects of captive breeding in the Australian aquaculture population; consequently, the effect of sampling bias cannot be excluded. However, the mt*COI* signal of reduced diversity in South Africa is consistent with an introduced population and both mt*COI* and SNP‐based RASP analyses recovered Australian samples in a basal position, suggesting Australia as the plausible origin. This highlights the need for precautionary conservation efforts to prevent further spread.

## Graphical abstract

The genetic patterns of the bait polychaete, *Diopatra aciculata*, were explored using mt*COI* sequences and ddRAD‐derived SNPs from Australian and South African populations. Analyses revealed low SNP diversity in both regions, reflecting a bottleneck and a strong continental differentiation, with reduced mt*COI* diversity in South Africa, indicating a likely introduction from Australia. These results highlight the need for management to limit further spread in South African estuarine ecosystems.

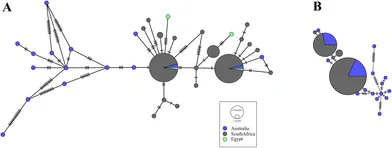

## 1 Introduction

The presence of a potentially introduced invasive species in a protected region, like the Knysna Estuary in South Africa, has important management implications. If the species is indigenous, research findings can contribute to the conservation of an important resource and protection from over‐exploitation. Conversely, if the species is alien and invasive, the information can be used to control or manage its further spread. Natural gene flow of marine organisms is influenced by the dispersal potential of various life stages, biogeographical barriers, and oceanic currents (Clegg and Phillimore 2010; Templeton 2019). More specifically, the population genetic structure of mobile, sessile, sedentary, and discreetly mobile marine benthic species is predominantly shaped by larval development, duration of the planktonic larval phase, and larval migration (Levin 2006; Woodin et al. 2014). Species with extended larval dispersal stages generally show limited geographic structure due to higher levels of gene flow among distant populations (Cowen et al. 2007; Coleman et al. 2013; Smith et al. 2015; Kara 2019), while species with limited vagility often show higher levels of geographic structure (Teske et al. 2007; Dibacco et al. 2006).

The tube‐building polychaete, *Diopatra aciculata* Knox and Cameron 1971, an ecosystem engineer (Woodin 1978; Berke 2022), was originally described from the estuaries of New South Wales, Victoria, South Australia, and Western Australia (Paxton 1993; Figure 1A) and is commercially farmed in New South Wales (Gambi et al. 1994; Safarik et al. 2006; Cole et al. 2018). The species is considered one of the five most expensive bait polychaete species worldwide (Watson et al. 2017) and in South Africa (where it is known colloquially as the estuarine moonshine worm), it is harvested along the south coast (Simon et al. 2019, 2022; figure 1C). The first *Diopatra* species reported along the South African shore was *D. cuprea* (Bosc 1802) in the Cape in 1908 (Von Carl Chun et al. 1908), and then *D*. *neapolitana* (Delle Chiaie 1841) from the Knysna Estuary in the 1940s (Day et al. 1951) and Swartkops Estuary in the 1950s (Macnae 1957). A few decades later, *D. neapolitana* was reported as bait in the Swartkops Estuary in the 1970s (Van Der Westhuizen and Marais 1977), followed by a report of a *Diopatra* sp. from Knysna in the 2000s (Napier et al. 2009). However, in 2018, *D. neapolitana*, previously reported from the Knysna and Swartkops Estuaries, was correctly identified as *D. aciculata* (Elgetany et al. 2020; van Rensburg et al. 2020), and is now the second most popular polychaete bait species in the Knysna Estuary (Simon et al. 2019). More recently, *D. aciculata* was reported for the first time in the Keurbooms Estuary (reported incorrectly as *D. cuprea* ; De Souza 2021; van Rensburg 2025) and in Egypt (Elgetany et al. 2020; Figure 1B).

**FIGURE 1:**
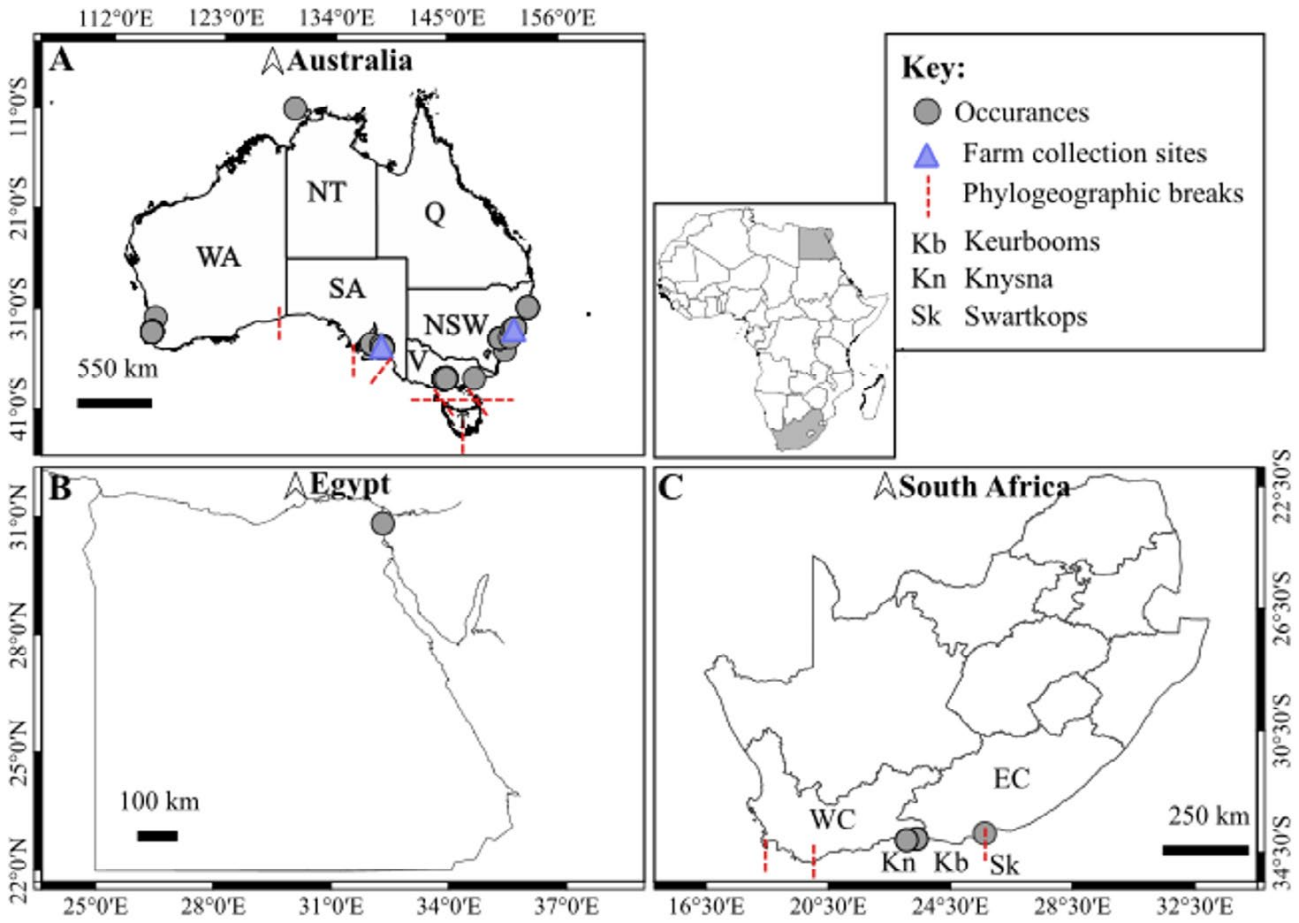
Global distribution of *Diopatra aciculata*, with each circle representing a record. (A): Australia. Samples were obtained from an aquaculture farm in Australia (blue triangles represent the locations where broodstock were collected). (B): Egypt. (C): The three South African estuaries sampled included Knysna (Kn), Keurbooms (Kb), and Swartkops (Sk). Key: NSW, New South Wales; NT, Northern Territory; Q, Queensland; SA, South Australia; T, Tasmania; V, Victoria; WA, Western Australia. EC, Eastern Cape; WC, Western Cape; Dotted lines: Known phylogeographic breaks (Australia (see Teske et al. 2017); South Africa from left to right: Cape Point; Cape Agulhas; Algoa Bay (see Teske et al. 2011)).

The natural range of *D. aciculata* is uncertain (Elgetany et al. 2020) as the first reports of its presence in South Africa (Day et al. 1951; Macnae 1957) predate the original description of the species in Australia (Knox and Cameron 1971). However, given the wider distribution in Australia (Figure 1A), it is more plausible that *D. aciculata* is not native to South Africa. A preliminary molecular investigation of 31 *D. aciculata* specimens collected in Australia, Egypt, and South Africa revealed shared mitochondrial DNA haplotypes, indicating either natural genetic connectivity among continents or dispersal facilitated through human activities (Elgetany et al. 2020). Due to the small sample size in this study, the native range could not be determined and *D. aciculata* was therefore classified as cryptogenic (Elgetany et al. 2020; van Rensburg et al. 2020). Currently, no evidence is available for the natural dispersal of *Diopatra aciculata* but given that this species produces lecithotrophic larvae with a short planktonic stage (4 to 6 days; Paxton and Safarik 2008), it is hypothesised that some level of small‐scale natural dispersal is possible. Evidence of dispersal via associations with shellfish aquaculture (Woodin et al. 2014) and the sale of live fishing bait (Dağli et al. 2005; Saito et al. 2014) has been documented for members of the genus with similar life histories (discretely motile, free‐spawning, and lecithotrophic larvae with a short planktonic stage). However, these pathways are unlikely to explain a putative introduction to South Africa. In contrast, the larvae of some polychaetes, such as Spionidae, are very common in ship ballast water (Carlton and Geller 1993), suggesting that ballast is a more likely vector even though *Diopatra* spp. larvae have not been recorded from ballast water samples to date. However, long‐distance natural dispersal for *D. aciculata* (particularly across ocean basins) is highly unlikely, and any signatures of intercontinental gene flow among populations are more plausibly a result of human‐mediated dispersal (Elgetany et al. 2020).

Given the lack of conclusive evidence from previous mitochondrial DNA (mtDNA) studies (Elgetany et al. 2020), a more in‐depth approach is required; therefore, this study aimed to investigate the genetic diversity of *D. aciculata* to determine the native range using a dual marker approach, namely sequences of the mt*COI* gene fragment and genome‐wide single‐nucleotide polymorphisms (SNPs) generated through ddRAD sequencing. Given the fragmented distribution among South African estuaries, the distance between Australia and South Africa (> 13,000 km), and the limited dispersal potential of *D. aciculata* (up to 50 km; Woodin et al. 2014), it is hypothesised that there is no natural gene flow between South African and Australian populations (see Carr et al. 2011). Furthermore, natural gene flow among the South African estuaries will be limited, with physical distance driving genetic differentiation among sampling sites.

## 2 Methodology

### 2.1 DNA Extraction, Amplification, and Sequencing

*Diopatra aciculata* samples were obtained from Australia (*n* = 17), South Africa (*n* = 180), and Egypt (*n* = 7; Figure 1). The Australian samples originated from a captive bait farm population in New South Wales (Australian Museum reference number: MI2023032). The aquaculture stock population was originally collected from Port Adelaide Harbour (South Australia) in the 2000s and Lake Macquarie (New South Wales) in the 2010s (Figure 1A; triangles) and has been bred on a single farm since (Aquabait Pty Ltd). Within South Africa, samples were collected from Knysna (*n* = 140), Keurbooms (*n* = 20), and Swartkops Estuaries (*n* = 20; Figure 1C). All samples were preserved and stored in 99% ethanol prior to DNA extraction.

Total genomic DNA was extracted from muscle tissue using a Quick‐DNA Miniprep Plus DNA extraction kit (Zymo Research) according to the manufacturer's instructions. The DNA integrity was assessed through gel electrophoresis (1% agarose gel), and concentration was determined using a Qubit broad‐range double‐stranded DNA assay (Invitrogen). The extracted DNA was stored at −20°C until further processing.

The mt*COI* gene fragment was amplified using the universal mt*COI* LCO and HCO primers (Folmer et al. 1994). The PCR thermal cycling conditions were as follows: 95°C for 3 min, followed by 35 cycles of 94°C for 1 min, 45°C for 30 s, and 72°C for 2 min. This was followed by a final extension at 72°C for 7 min and a subsequent cooling to 12°C. PCR products were purified and sequenced using Sanger sequencing with the BigDye Terminator v3.1 sequencing kit (Thermo Fisher/Applied Biosystems) on an ABI 3500xl or ABI 3730xl sequencer at the Central Analytical Facility of Stellenbosch University.

### 2.2 mtDNA Phylogenetic and Phylogeographic Analyses

All mt*COI* gene sequences were manually edited in BioEdit v.7.2.6 (Hall 1999). A BLASTn search was performed to find similar mt*COI* sequences for the species and genus (*e*‐value threshold: *E* ≤ 10^−5^). These sequences were downloaded (accession numbers in Table S1), combined with our sequence data and aligned in MEGA11 (Tamura et al. 2021) using the ClustalW algorithm (Larkin et al. 2007), with default parameters. The final alignment was trimmed to equal lengths across all sequences for downstream analyses. DnaSP 6 (Rozas et al. 2017) was used to determine the total number of haplotypes (*H*), haplotype diversity (*h*), and nucleotide diversity (*π*).

Genetic distances were calculated using MEGA11. Analyses were conducted using the Maximum Composite Likelihood model selected based on the BIC score. Using the same model, a Bayesian Inference tree was constructed in MrBayes v3.2 (Ronquist et al. 2012) with four parallel Markovian Chains run for 90,00,000 generations, sampling every 1000th tree. The first 25% of trees were discarded as burn‐in, and the remaining trees were used to construct a 50% majority‐rule consensus tree illustrating the Posterior Probability (PP) support for each node. Tracer v 1.7.2 (Rambaut et al. 2018) was used to assess the effective sample sizes (ESS) for all parameters and the trees were visualised in FigTree v1.4.4 (http://tree.bio.ed.ac.uk/software/figtree). *Lysidice natalensis* Kinberg, 1865 (Order: Euncida; Zanol et al. 2021) was used as the outgroup. Based on the above results, only sequences forming part of the monophyletic *D. aciculata* clade were used for further analyses.

POPART (Leigh and Bryant 2015) was used to produce a statistical parsimony haplotype network (Bandelt et al. 1999) of all the *D. aciculata* specimens. Furthermore, a haplotype network consisting of a subset of 18 randomly selected sequences from Australia and South Africa was constructed using the R packages *ape* (Paradis et al. 2004) and *pegas* (Paradis and Barrett 2010; iterations: 1000), to account for uneven sampling between the regions. The divergence between South Africa and Australia and between the South African estuaries was investigated using an AMOVA computed in ARLEQUIN v 3.5.2.2 (Excoffier et al. 2005) and tested for significant differentiation using 1000 permutations. To compare populations using equal sample sizes, the *D. aciculata* dataset was randomly subsampled 20 times using the R packages *ape* (Paradis et al. 2004) and *pegas* (Paradis and Barrett 2010), with each replicate including 10 randomly selected sequences from Australia, Knysna, Keurbooms, and Swartkops, and four sequences from Egypt. A Bayesian inference tree was constructed for each replicate as described above (Table S2), using *D. neapolitana* as an outgroup. The resulting topologies were compared to assess the robustness of the inferred relationships; the replicate with posterior support most similar to the median across replicates was selected as the majority‐rule consensus topology moving forward. The ancestral geographic ranges at each node were reconstructed using Bayesian Binary MCMC (BBM) analysis implemented in RASP v4.0 (Yu et al. 2020). Geographic ranges were coded as follows: A, Australia; B, Keurbooms; C, Knysna; D, Swartkops; E, Egypt.

### 2.3 Whole‐Genome Sequencing, Library Preparation, and Single‐Nucleotide Polymorphism Identification

To provide a more sensitive means of contemporary fine‐scale population structure, whole‐genome SNPs were identified. Briefly, a total of 86 samples from Australia (*n* = 13), Knysna (*n* = 51), Keurbooms (*n* = 11), and Swartkops (*n* = 11) that passed the quality control criteria (> 300 ng DNA and 10 ng/ul) were randomly selected and shipped to CD Genomics (New York City, USA) for double‐digest restriction‐site associated DNA (ddRAD) sequencing. The genomic DNA of each sample was digested using restriction enzymes *DpnII* and *MspI* and incubated at 37°C for 5 h. The fragmented DNA was then purified with AMPureXP beads (Agencourt) to remove the non‐target length fragments and then ligated to the barcoded adapters. The eluted fraction was amplified with oligo primers that introduced TruSeq indexes and subsequently bead purified. Size selection (~220 bp–450 bp) was done on a BluePippin instrument (Sage Science Inc.). The resulting libraries were checked with both a Qubit 2.0 Fluorometer (Invitrogen, Carlsbad, Canada) and a Bioanalyzer DNA assay (Agilent Technologies, Santa Clara, Canada). Samples with different barcodes were pooled in equal concentrations on multiplexing batches based on the Qubit concentration. The constructed libraries finally underwent paired‐end sequencing on an Illumina NovaSeq6000 platform. Demultiplexing was performed using fastp (Chen et al. 2018). The quality of the raw data was evaluated using FastQ v0.12.1 (Wingett and Andrews 2018).

Due to the lack of a suitable reference genome, SNPs were identified using the *de novo* Stacks v2.59 (Catchen et al. 2013) pipeline with default parameters (‐m 3; ‐M 2;‐p 2; ‐N4; ‐r 0.80). The identified SNPs were filtered using PLINKv2.0 (Purcell et al. 2007): all variants were required to have a minor allele frequency (MAF) of 0.05 and missing call rates (geno) exceeding 0.2 were removed. To identify outlier samples, analyses were conducted using the R package *OutFLANK* (Whitlock and Lotterhos 2015).

### 2.4 SNP Data Analyses

The *populations* package in Stacks v2.59 was used to determine the nucleotide diversity (*π*), observed heterozygosity (*H*_O_), expected heterozygosity (*H*_E_), inbreeding coefficient (*F*_IS_), and the mean frequency of the most frequent allele at each locus (*P*) for each population. Pairwise Φ_ST_ for each geographic region (South Africa and Australia) was calculated using the R package *dartR* v2 (Mijangos et al. 2022) with 1000 permutations. The divergence between South Africa and Australia was investigated using an AMOVA in the R packages *vcfR* (Knaus and Grünwald 2017) and *poppr* (Kamvar et al. 2014). A Mantel test was performed using the R package *dartR* v2 (Mijangos et al. 2022) with 999 permutations to investigate signals of isolation by distance between the South African populations (distance inferred using Google Earth Pro).

To visualise clustering patterns between the Australian and South African populations, a Discriminant Analysis of Principal Components (DAPC) was performed using the *adegenet* package in R (Jombart and Ahmed 2011). The *find*.*clusters* function was used to determine the number of clusters, with cross‐validation (30 replications testing a principal component range from one to 40) determining the optimal number of principal components to retain. Next, the lowest Bayesian information criterion (BIC) value was chosen (Jombart et al. 2010) as the slope begins to plateau to select the optimal *K* value (*K*: 1–10).

A likelihood tree was constructed using the packages *ape* (Paradis et al. 2004) and *phangorn* (Schliep 2011) in R (bootstrap: 1000). The best model was determined using the modeltest function, and it was selected based on the lowest BIC value (model selected: TVMe+G(4)). The ancestral geographic ranges at each node were reconstructed using Bayesian Binary MCMC (BBM) analysis in the program RASP s v4.0 (Yu et al. 2020). The distributional ranges were A (Australia); B (Knysna & Keurbooms); C (Swartkops); and D represents *D. neapolitana* from Egypt as the outgroup.

## 3 Results

### 3.1 mtDNA Phylogeny

The mt*COI* gene fragment analyses were based on a 600 base pair alignment consisting of 169 sequences from 12 *Diopatra* species (Table S1; model selected: GTR+Gamma+I). The Bayesian phylogeny of the mt*COI* gene fragment for the *Diopatra* genus revealed several clades with moderate to high nodal support (Figure 2), supporting the high level of differentiation among the species in this genus (Table S3). *Diopatra aciculata* from Australia and South Africa (Keurbooms, Swartkops, and Knysna) formed a monophyletic clade distinctly divergent from the other species in the genus (Figure 2; Figure S1).

**FIGURE 2:**
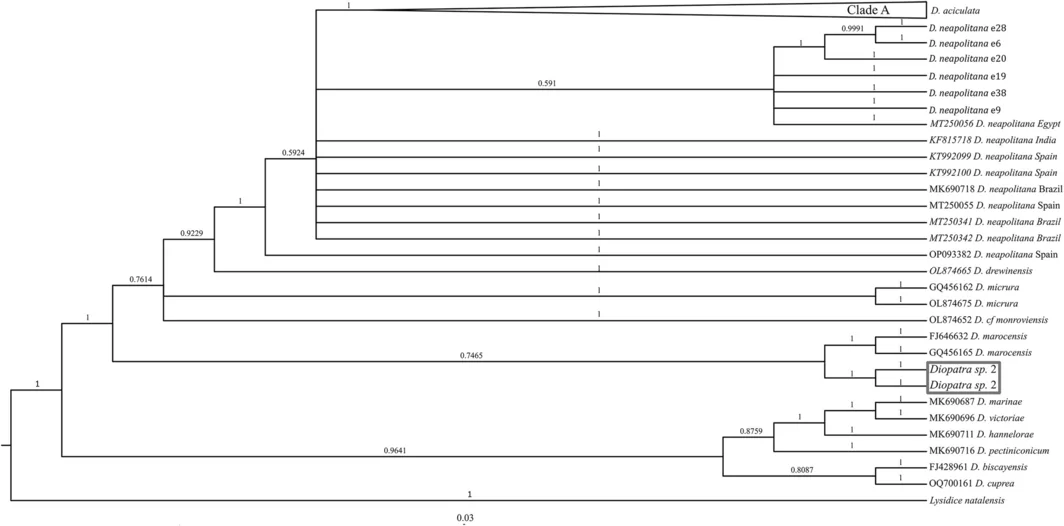
Bayesian 50% majority‐rule consensus tree based on the *mtCOI* gene of the genus, including publicly available sequences from GenBank (denoted with accession numbers before the species name). Clade A represents all the *D. aciculata* sequences. The box represents an additional *Diopatra* species collected in Knysna Estuary, clustering with *D. marocensis*. The tree is rooted with *Lysidice natalensis* (Order: Eunicida). Support for clades: Posterior probabilities (Bayesian likelihood).

The seven Egyptian *Diopatra* specimens collected in this study clustered with *D. neapolitana*, weakly supported by Bayesian analyses (0.53; Figure 2). Furthermore, two samples morphologically identified as *D. aciculata* collected from the mouth of the Knysna Estuary grouped closely with *D. marocensis* Paxton et al., 1995 with significant and strong support from the Bayesian analyses (0.99). These specimens were excluded from downstream analyses.

### 3.2 mtDNA Diversity and Structure

Analyses of *D. aciculata* were based on 139 mt*COI* sequences from three countries: South Africa (117), Australia (18) and Egypt (4) (Table S1). The dataset displayed a moderately high haplotype diversity and low nucleotide diversity (Table 1). Haplotypic and nucleotide diversity were much higher in the Australian population compared to the South African population. (Table 1). This result was further substantiated when the dataset was subsampled to equal sizes (Table 1). Within South African estuarine populations, the Knysna individuals had higher genetic haplotypic diversity compared to those in Keurbooms and Swartkops (Table 1). Haplotypic and nucleotide diversity were much higher in the Australian population compared to the South African population (Table 1).

**TABLE 1:** Summary of the *COI* molecular diversity for each *D. aciculata* population and sub‐population in this study.

	Sample size	No. of haplotypes	Private haplotypes	Subset to 18 samples	S	*k*	*H*_d_	*π*
South Africa								
Knysna	93	17	13	—	23	2.202	0.685	0.00369
Keurbooms	10	3	1	—	7	2.400	0.644	0.00400
Swartkops	14	3	0	—	4	1.835	0.648	0.00306
Total	117	18	16	*H*_d_: 0.691	27	2.202	0.690	0.00367
				(*π*: 0.004)				
Australia	18	16	13	*H*_d_: 0.987 (*π*: 0.016)	34	9.575	0.987	0.01596
Egypt	4	4	2	—	11	6	1	0.01000
Total	139	34	31	—	43	3.432	0.746	0.00572

*Note:* S: Number of segregating (polymorphic/variable) sites; *k*: Average number of pairwise nucleotide differences; *H*_d_: Haplotype diversity; *π*: Nucleotide diversity.

Significant population differentiation was observed between Australia and South Africa (Table 2). However, no population differentiation was observed between any of the South African populations (Knysna and Keurbooms: Φ_ST_ = 0.00; *p* > 0.05; Keurbooms and Swartkops: Φ_ST_ = 0.032; *p* > 0.05; Swartkops and Knysna: Φ_ST_ = 0.032; *p* > 0.05). The haplotype network of *D. aciculata* revealed a total of 34 haplotypes with two common haplotypes shared among the Australian, South African and Egyptian populations (Figure 3A). Samples from both Australia and South Africa also displayed a relatively large number of private haplotypes (Australia: 13; South Africa: 16; Figure 3A). Conversely, the subset of 18 randomly selected samples from Australia and South Africa revealed two shared and 14 and three private haplotypes, respectively (Figure 3B). Australian haplotypes occupied basal positions in 16 of 20 subsampled phylogenies (Figure S2). The mt*COI*‐based RASP analysis recovered the Australian lineage (A) as deeply basal relative to the populations from South Africa and Egypt (Figure 4A).

**TABLE 2:** Analysis of molecular variance (AMOVA) of the *COI* gene and SNP dataset between the Australian and South African populations.

Source of variation	mt*COI*				SNPs			
	(*p* < 0.05*; Φ = 0.316)				(*p* = 0.01*; Φ = 0.139)			
	df	Sum of squares	Σ	%	df	Sum of squares	Σ	%
Among groups	1	36.279	1.028	31.58	1	565.417	19.996	13.88
Within populations	133	296.278	2.228	68.42	84	10424.834	124.105	86.12
Total	134	330.585	3.256	100.00	85	10990.251	144.101	100.00

*Note:* *: Statistically significant; Σ: Variance components; %: Percentage variation.

**FIGURE 3:**
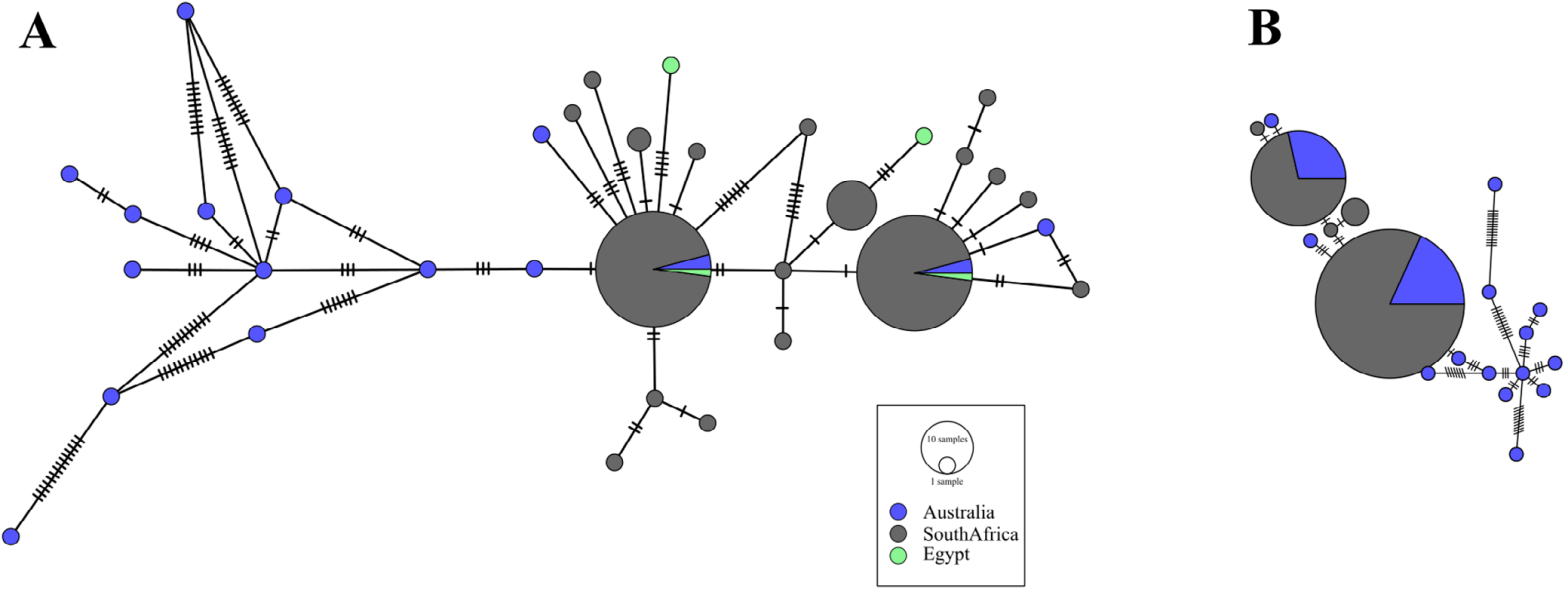
Minimum spanning network (Epsilon = 0) of the mt*COI* gene of *D. aciculata* specimens collected in South Africa and Australia. The size of the circles represents the number of individuals that belong to each haplotype, while the dashes represent the number of mutational steps between each haplotype. (A) The complete *D. aciculata* mt*COI* dataset; (B) A subset of 18 randomly selected sequences from Australia and South Africa, respectively (replicated 1000 times).

**FIGURE 4:**
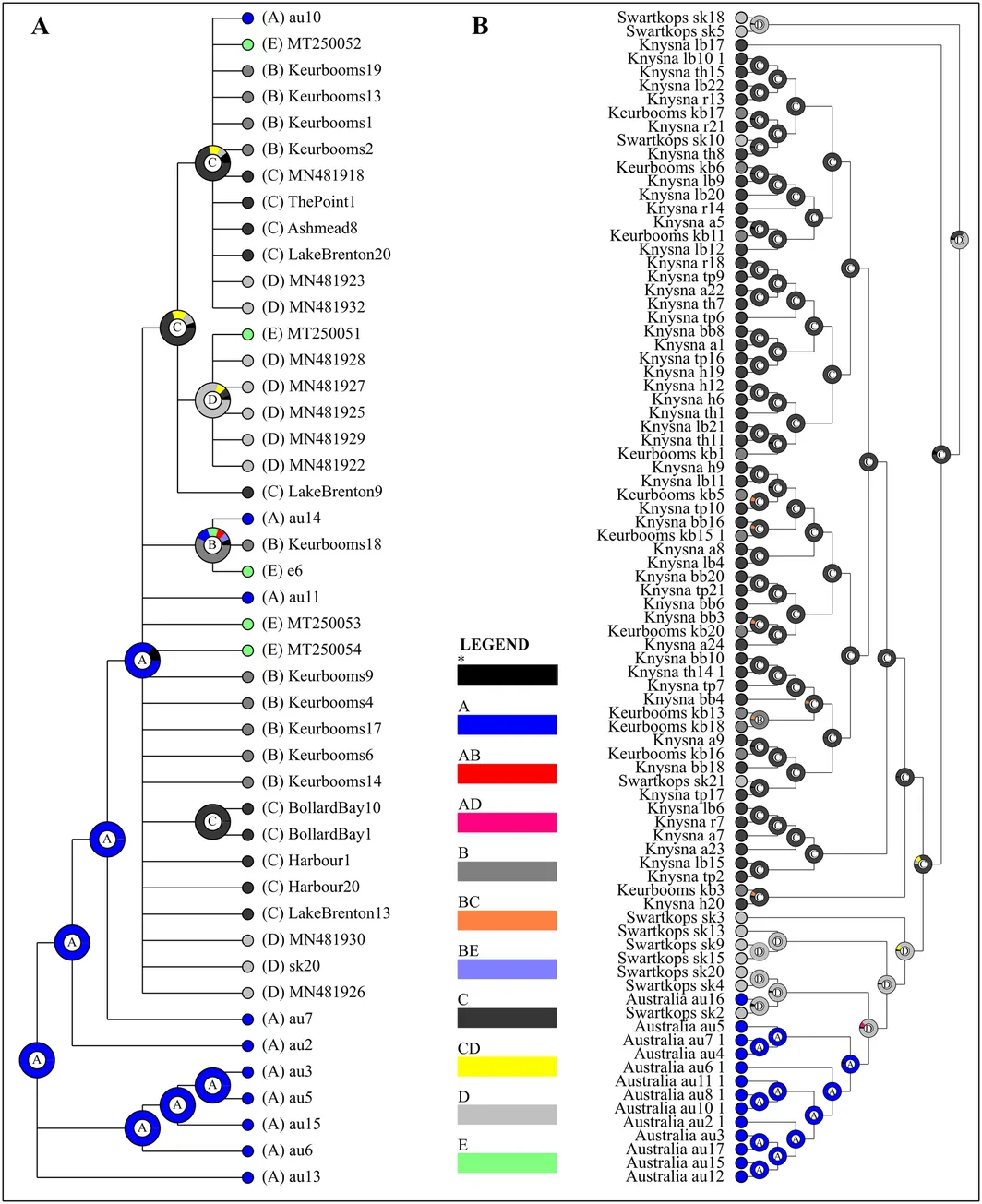
Ancestral range reconstruction of *Diopatra aciculata* inferred using Bayesian Binary MCMC (BBM) analysis implemented in RASP. (A) Mt*COI* phylogeny based on a subset of 10 sequences per sampling location. (B) SNP‐based phylogeny. Pie charts at internal nodes represent the relative probabilities of inferred ancestral geographic ranges. Trees were rooted using *Diopatra neapolitana*. Geographic regions are indicated as follows: A, Australia; B, Keurbooms; C, Knysna; D, Swartkops; and E, Egypt.

### 3.3 Nuclear Diversity and Differentiation

Double‐digest restriction‐site associated DNA sequencing yielded 1,578,201,490 paired‐end reads, and initial SNP identification resulted in 1,480,741 SNPs from 86 samples. Clean‐up and filtering yielded 1619 SNP loci from these specimens. The farmed Australian population displayed the lowest % polymorphic loci, low expected heterozygosity, low nucleotide diversity and the lowest inbreeding coefficient (Table 3). Higher diversity statistics were observed in the wild South African populations: Knysna, Keurbooms, and Swartkops (Table 3). Supporting the mt*COI* findings, significant but moderate population differentiation was observed between Australia and South Africa (Table 2). On a global scale, six times more variation was attributed to within populations than between populations (Table 2; AMOVA; *p* = 0.01). Additionally, significant population differentiation was documented between Swartkops and Knysna (*F*_ST_ = 0.04629; *p* < 0.05) and Swartkops and Keurbooms (*F*_ST_ = 0.04632; *p* < 0.05), but no population differentiation was documented between Knysna and Keurbooms (*F*_ST_ = 0.00; *p* < 0.05). The DAPC yielded results like those of the AMOVA. The optimal number of DAPC clusters (Figure 5A), as determined by the lowest BIC value, was *K* = 4, but due to the similarly low BIC, *K* = 3 was also explored (Figure 5B,C). The clustering patterns of *K* = 4 align with the populations of *D. aciculata* sampled: Australia, Swartkops, Knysna and Keurbooms Estuaries. Distinct clusters are seen for Australia and the Swartkops Estuary, respectively, while the Knysna and Keurbooms Estuaries cluster more closely together (Figure 5A,B). The Mantel test revealed a non‐significant but strong positive correlation (Mantel test; *r* = 0.9905, *p* > 0.05) between the genetic and geographic distance among the South African populations.

**TABLE 3:** Genetic diversity estimates for 1619 SNP loci from 86 *Diopatra aciculata* individuals from various populations.

	*n*	Polymorphic loci (%)	*p*	*H*_O_	*H*_E_	*π*	*F*_IS_
South Africa							
Knysna	51	0.93598	0.99802	0.00254	0.00280	0.00283	0.00094
Keurbooms	11	0.84380	0.99810	0.00241	0.00265	0.00280	0.00101
Swartkops	11	0.93430	0.99794	0.00251	0.00290	0.00306	0.00147
Total	73	0.73848	0.99810	0.00235	0.00265	0.00215	0.00067
Australia	13	0.58936	0.99867	0.00172	0.00184	0.00192	0.00054

*Note:* *n*: Sample number; *p*: Mean frequency of the most frequent allele at each locus in this population; *H*_O_: Observed heterozygosity; *H*_E_: Expected heterozygosity; *π*: Nucleotide diversity; *F*_IS_: Inbreeding coefficient.

**FIGURE 5:**
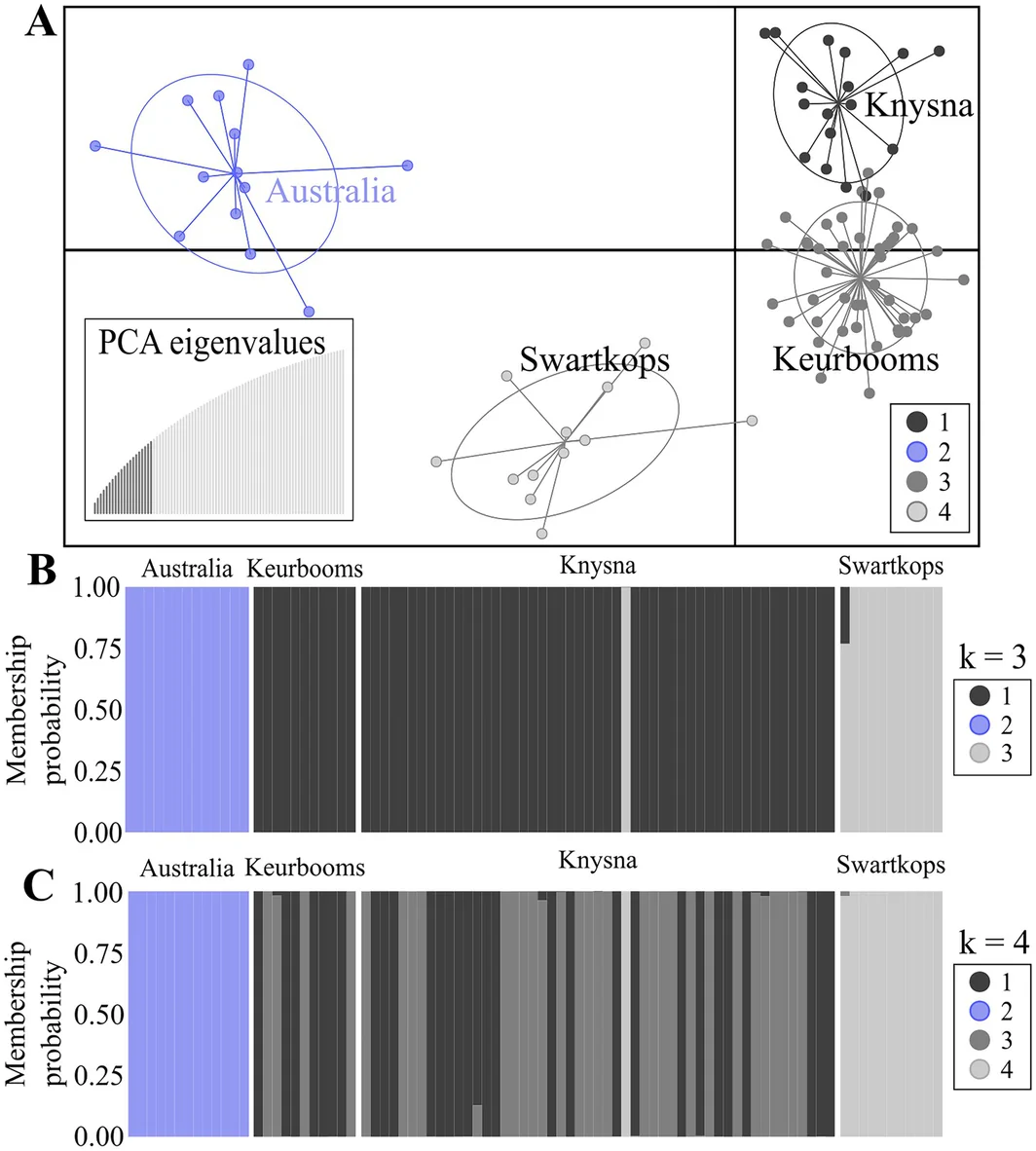
Genetic structure of *Diopatra aciculata*. (A) The discriminant analysis of principal component (DAPC) of 1619 genome‐wide SNP profiles from 86 individuals across four populations. Dots represent individuals, bounded by a 95% confidence circle. (B) and (C) The admixture proportions of the four populations of *D. aciculata* were assigned using DAPC (*adegenet*) (B: *K* = 3; C: *K* = 4). The x‐axis is membership probability, and each vertical bar represents a sample.

The SNP‐based RASP analysis recovered the Australian lineage as deeply basal relative to the South African samples (Figure 4B). Within South Africa, Swartkops‐associated (D) individuals also occupied early‐diverging positions in the phylogeny, whereas the more derived clades were dominated by extensive diversification among individuals from Knysna and Keurbooms. Ancestral range reconstructions at deeper nodes were primarily associated with Australia (A) and mixed South African range states, while many internal nodes within the South African radiation were reconstructed predominantly as Knysna‐associated (C) ancestral states.

## 4 Discussion

The present study highlights the difficulties in determining the origin and subsequent spread of potentially invasive bait species like *Diopatra aciculata*, with conflicting signals between the mt*DNA* and the SNP datasets further deterring firm conclusions. Nonetheless, (i) the wider distribution record for the species in Australia, (ii) the lower mt*DNA* genetic diversity for the South African population, (iii) the sharing of two common mt*DNA* haplotypes between South Africa and Australia, and (iv) the significant population differentiation between these two continental populations all point to a scenario that *D. aciculata* is likely an invasive species, probably introduced from Australia, and has the potential to spread among estuaries in South Africa.

### 4.1 Native Range and Genetic Diversity

The type locality of a species provides important historical context, not only for its description but also for early occurrence records. This, however, does not necessarily reflect its true native range (Pettengill et al. 2007). In fact, studies often reveal that the location of the first description differs from the native range (e.g., Carlton 2009; Radashevsky et al. 2023a, 2023b). Although circumstantial, patterns of genetic diversity and population structure of native and invasive species are predicted to be different (Xia et al. 2020). Native populations usually exhibit a higher genetic diversity than invasive populations (Rollins et al. 2013; Yi and Kim 2020) and the population structure of invasive populations is shaped by the nature of the founder population (including the possibility of multiple introductions through time). Along these lines, genetic diversity can also offer insight into the frequency and number of individuals introduced during colonisation events, as well as the pathways through which invasions occurred (Barrett 2015; Williams et al. 2016; Malan et al. 2020; Radashevsky et al. 2023a, 2023b). From genetic diversity estimates, predictions are also made regarding the capacity of a species to adapt to new and changing environments (Hiesey 1966; Sakai et al. 2001; Drake and Lodge 2006; Crawford and Whitney 2010). The higher mtDNA genetic diversity documented in Australia is likely a result of a species in its natural range and substantial geographic distance. Conversely, the genome‐wide SNP dataset revealed low genomic diversity that is more consistent with captive breeding (Mignon‐Grasteau et al. 2005), resulting from intense micro‐evolutionary forces (inbreeding, drift and selection; Hull et al. 2024). The elevated mtDNA diversity and comparatively low SNP‐derived *F*_IS_ observed in the Australian aquaculture population likely reflect its mixed demographic origin rather than natural population processes. According to aquaculture records (Aquabait pers. comm.), the captive stock was established using individuals sourced from both South Australia and New South Wales, regions separated by substantial geographic distance and multiple recognised phylogeographic barriers (see Teske et al. 2017 for review; figure 1A). The combination of historically differentiated source populations would be expected to inflate mitochondrial diversity through the introduction of divergent maternal lineages (Lynch 2023), while admixture among previously structured populations would increase nuclear heterozygosity and reduce apparent inbreeding coefficients through isolate breaking. Consequently, the genetic patterns observed in the Australian samples are most parsimoniously explained by anthropogenic translocation and admixture associated with aquaculture practices. Both the mt*COI* and SNP‐based RASP analyses recovered Australian samples in a basal position relative to the South African populations, potentially indicating an early divergence between Australian and South African lineages. While suggestive of Australia as the origin/native range of the species, this interpretation should be treated cautiously, as the Australian samples were derived from an aquaculture population established through the admixture of individuals sourced from geographically distinct locations. Furthermore, the increased structuring seen in the Australian clade is likely due to the monthly spawning cycle exhibited by the farmed population compared to the annual cycle documented in the Knysna Estuary (Schoeman 2025). The much lower mtDNA and low genomic diversity documented in the South African populations are more characteristic of introduction event/s along the South African coastline (Roman and Darling 2007; Williams et al. 2016), further substantiated by shared haplotypes between Australia and South Africa (Elgetany et al. 2020). The farmed Australian population displayed a higher haplotypic and nucleotide diversity compared to the wild South African populations. It is important to note that the many mt*COI* haplotypes detected in the South African populations may be attributed to the large sample size discrepancy (~ sixfold; see Subramanian 2016). While higher genetic diversity is often indicative of the native range, assigning Australia as the native range remains most plausible, especially given our sampling from an aquaculture facility that sourced individuals from two geographically distinct locations. To resolve this uncertainty, future studies should include samples from wild populations in Australia.

### 4.2 Population Structure

#### 4.2.1 Shared Ancestry Between Australia and South Africa

Cosmopolitan distributions have been documented in some polychaete species; however, such cases are typically associated with deep‐sea taxa that exhibit wide distributions and are especially uncommon in estuarine species (see Hutchings and Kupriyanova 2018). *Diopatra aciculata* collected from Australia and South Africa are genetically isolated and show little evidence for recent gene flow (Hupało et al. 2023; Choquet et al. 2023). The shared mitochondrial haplotypes can likely be attributed to a recent introduction event (Simon et al. 2009; Williams et al. 2016; Elgetany et al. 2020), and the large number of private haplotypes found in the South African population may indeed reflect multiple successive introductions or a single introduction from a genetically diverse source population not sampled in the present study (Kolbe et al. 2004; Roman and Darling 2007; Williams et al. 2016). However, it must be noted that the robustness of this comparison is limited by the available data. The complexity of the founder event/s likely contributes to the pattern of genetic distinctness detected between the Australian and South African populations by both the mt*DNA* and the genome‐wide SNP analyses (Chieu et al. 2023). *Diopatra* species are primarily transported through the shipment of live bait (Dağli et al. 2005; Saito et al. 2014) and aquaculture (Woodin et al. 2014). However, there is no record of live polychaete worms having been imported into South Africa (Department of Forestry, Fisheries and the Environment 2017), and aquaculture, although possible, is also unlikely. The first exotic oyster spats of *Ostrea edulis* Linnaeus, 1758 and *Crassostrea angulata* Lamarck, 1819, were imported from Europe in the 1950s (Korringa 1956). After several failed aquaculture attempts, *Magallana gigas* (Thunberg, 1793) spat was imported from France, England, and Chile in 1973 (Korringa 1956; Haupt et al. 2010). Not only were these several years after the first report of *D. aciculata* in the region, but no documented spat was ever imported from Australia (see Haupt et al. 2010). Although polychaete species have been transported via hull fouling (Pettengill et al. 2007; Fitridge et al. 2012), it is unusual for large, discretely mobile sand‐tube dwelling species, such as *D. aciculata* , to follow this pathway. The free‐floating larvae of *Diopatra* are also unlikely to survive in ballast water during transit due to their short planktonic phase (Berke et al. 2010). However, several studies have detected polychaetes (Williams et al. 1988; Tepolt et al. 2020), including living onuphids (Coutts et al. 2003) in ballast sediment. Knysna Estuary in particular, acted as an active port during the 1800s and 1900s, regularly receiving ships using solid ballast (162 vessels from 1817 to 1837; Muller 1985; Hart and Halkett 1998), suggesting that shipping could have acted as the vector for *D. aciculata* into South African estuaries.

Members of the genus *Diopatra* are notoriously difficult to identify (Paxton 2002; Arias and Paxton 2015), as is evident by the two unidentified *Diopatra* specimens collected at the mouth of the Knysna Estuary. Additionally, although Elgetany et al. (2020) recorded and sequenced four *D. aciculata* from the Suez Canal in Egypt, the specimens collected in this study were genetically grouped with *D. neapolitana*, highlighting the challenges of species identification within the genus.

#### 4.2.2 Sub‐Structuring in South Africa

The isolated nature of estuaries leads to a high likelihood of genetic isolation among populations (Bilton et al. 2002; Teske et al. 2006; Simon et al. 2020; Tepolt et al. 2020), especially in the absence of stepping‐stone populations (Duforet‐Frebourg and Slatkin 2015). The mt*COI* analysis revealed shared haplotypes and significant differentiation of Swartkops from Keurbooms and Knysna Estuaries, respectively. These patterns closely resemble those identified in *Arenicola loveni* Kinberg 1866, a native polychaete with similar ecological characteristics and life history (Simon et al. 2020). However, unlike *D. aciculata* , *A. loveni* has a wider distribution with multiple stepping‐stone populations in estuaries and beaches along the same coastal region (Simon et al. 2020). Given the lack of stepping‐stone populations, especially between Knysna and Swartkops Estuaries (van Rensburg 2025), the pattern observed for *D. aciculata* in South African populations could indicate human‐mediated movement of individuals among estuaries (Williams et al. 2016). The more fine‐scale SNP data revealed non‐significant population differentiation between the Knysna and Keurbooms Estuary (Garden Route region; ~20 km apart), similar to other estuarine onuphid species (Padovan et al. 2020; Ziegler et al. 2025). However, significant population differentiation was evident when these two populations were compared to the Swartkops Estuary (~230 km apart). Additionally, the SNP‐based RASP analysis indicates divergence of Swartkops from the Knysna and Keurbooms populations, suggesting substantial regional diversification along the southern South African coast. Similar small‐scale isolation by distance, where genetic differentiation increased with geographic distance, has been observed in *D. cuprea* (Ziegler et al. 2025), which can disperse naturally up to 50 km in their native range in the United States (Woodin et al. 2014). Furthermore, *D. aciculata* may be capable of greater dispersal; while it produces lecithotrophic larvae with a short planktonic phase (Paxton and Safarik 2008), the larger oocyte size observed in the Knysna Estuary (Schoeman 2025) is suggestive of a longer planktonic larval phase.

*Diopatra aciculata* was absent from earlier macrobenthic faunal surveys in Keurbooms Estuary (Heydorn et al. 1980), and first reported there in 2021 (De Souza 2021) suggesting that the species was recently introduced or is expanding into this estuary. Natural dispersal of *D. aciculata* across the short distance between Knysna and Keurbooms Estuaries is possible (Woodin et al. 2014); daily movement of 1000 to 2000 m^3^ of water per second into and out of Knysna Estuary (Largier et al. 2000) would facilitate the movement of larvae, increasing potential for natural dispersal. However, anthropogenic dispersal of *D. aciculata* must not be discounted; fishermen often move live bait between estuaries, which could, under the right circumstances, regenerate if discarded (Simon et al. 2019; Schoeman and Simon 2023). Thus, similar permanently open estuaries near invaded estuaries (such as Kromme Estuary, ~80 km west of Swartkops) could also be at risk of invasion.

## 5 Conclusion

This study provides compelling, albeit not conclusive, evidence that Australia is the native range of *D. aciculata* , and that the species should be categorised as invasive in South Africa, where it is spreading naturally and/or anthropogenically. This highlights the need for conservation efforts in South Africa: as an ecosystem engineer, *D. aciculata* poses a risk to the native ecosystems, necessitating the need for strategies including monitoring and early detection programs, and restrictions on bait collection and movement to mitigate the further spread of the species.

## Author Contributions

**Stephanie Schoeman:** data curation (lead), formal analysis (equal), investigation (equal), methodology (equal), writing – original draft (equal), writing – review and editing (equal). **Conrad A. Matthee:** methodology (equal), project administration (equal), supervision (equal), writing – review and editing (equal). **Kelvin Hull:** formal analysis (equal), validation (equal), writing – review and editing (equal). **Carol A. Simon:** funding acquisition (equal), project administration (equal), resources (equal), supervision (equal), writing – review and editing (equal).

## Ethics Statement

The authors have nothing to report.

## Conflicts of Interest

The authors declare no conflicts of interest.

## Supporting information


**Table S1:** ece374520‐sup‐0001‐Supinfo.docx. *COI* sequence details, including accession numbers, sampling location and reference included in this study.
**Table S2:** A summary of the MrBayes parameters for the subsetted datasets.
**Table S3:** Pairwise genetic distances of the mt*COI* gene of *Diopatra aciculata* used in this study.
**Figure S1:** A Bayesian 50% majority‐rule consensus tree based on the cytochrome oxidase subunit one (COI) gene of the *D. aciculata* (Figure 2: Clade A) collected in South Africa (Knysna, Keurbooms, and Swartkops Estuaries), Egypt and Australia. Support for clades: posterior probabilities (Bayesian likelihood).
**Figure S2:** The Bayesian 50% majority‐rule consensus trees of 20 randomly subsetted sequences based on the cytochrome oxidase subunit one (COI) gene of the *D. aciculata* collected in South Africa (*n* = 30; Knysna, Keurbooms, and Swartkops Estuaries), Egypt (*n* = 4) and Australia (*n* = 10). Support for clades: posterior probabilities (Bayesian likelihood). Number 10 was used for further analysis.

## Data Availability

Raw data and sequences are available on GenBank (http://www.ncbi.nlm.nih.gov/bioproject/1405539).
