# Supplementary material for: Genetic Diversity and Putative Origin of the Bait Polychaete, Diopatra aciculata Knox and Cameron (Annelida: Onuphidae), Occurring in South Africa
Source: Ecol Evol. 2026 Nov 6;16(11):e74520. doi: 10.1002/ece3.74520 (PMC13743407; doi:10.1002/ece3.74520)
Supplement: Supplementary file 1 [file ECE3-16-e74520-s001.docx]

**Genetic diversity and putative origin of the bait polychaete, *Diopatra aciculata* Knox and Cameron, 1971 (Annelida: Onuphidae), occurring in South Africa**

## **Supplementary information**

Table S1: *COI* sequence details, including accession numbers, sampling location and reference included in this study.

| **Species** | **GenBank accession**  **number** | **Location** | **Reference** |
| --- | --- | --- | --- |
| *D. aciculata* Knox and Cameron, 1971 | GQ497522 | Australia | (Zanol et al. 2010) |
| *D. aciculata* Knox and Cameron, 1971 | MT250050-MT250054 | Egypt | (Elgetany et al. 2020) |
| *D. aciculata* Knox and Cameron, 1971 | MN481922 - MN481932 | Swartkops, SA | (van Rensburg 2019) |
| *D. aciculata* Knox and Cameron, 1971 | MN481907 - MN481921 | Knysna, SA | (van Rensburg 2019) |
| *D. aciculata* Knox and Cameron, 1971 | AY838867 | Australia | Struck et al 2006 |
| *D. cuprea* Bosc, 1802 | OQ700161 | USA | (Sotka et al. 2023) |
| *D. marocensis* Paxton et al, 1995 | GQ456165 | Portugal | (Rodrigues et al. 2009) |
| *D. marocensis* Paxton et al, 1995 | FJ646632 | Portugal | (Rodrigues et al. 2009) |
| *D. hannelorae* Seixas et al, 2020 | MK690711 | Brazil | (Seixas et al. 2020) |
| *D. marinae* Seixas et al, 2020 | MK690687 | Brazil | (Seixas et al. 2020) |
| *D. micrura* Seixas et al, 2020 | GQ456162 | Portugal | (Pires et al. 2010) |
| *D. micrura* Pires et al, 2010 | OL874675 | Morocco | (Hektoen et al. 2022) |
| *D. pectiniconicum* Seixas et al, 2020 | MK690716 | Brazil | (Seixas et al. 2020) |
| *D. victoriae* Seixas et al, 2020 | MK690696 | Brazil | (Seixas et al. 2020) |
| *D. monroviensis cf* Augener, 1918 | OL874652 | Guinea | (Hektoen et al. 2022) |
| *D. neapolitana* Delle Chiaie, 1841 | KT992099 & KT992100 | Spain | (Arias et al. 2016) |
| *D. neapolitana* Delle Chiaie, 1841 | MT250056 & MT250055 | Egypt | (Elgetany et al. 2020) |
| *D. neapolitana* Delle Chiaie, 1841 | OP093382 | Spain | (Escobar-Ortega et al. 2022) |
| *D. neapolitana* Delle Chiaie, 1841 | MT250341 & MT250342 | Brazil | (Bergamo et al. 2019) |
| *D. neapolitana* Delle Chiaie, 1841 | MK690718 | Brazil | (Seixas et al. 2020) |
| *D. neapolitana* Delle Chiaie, 1841 | KF815718 | India | (Singh et al, 2013) |
| *D. biscayensis* Fauchald et al, 2012 | FJ428961 | France | (Berke et al. 2010) |
| *D. drewinensis* Augener, 1918 | OL874665 | West Africa | (Hektoen et al. 2022) |
| *Lysidice natalensis Kinberg, 1865* | MN419168 | South Africa | (Simon et al. 2021) |

**Table S2:** A summary of the MrBayes parameters for the subsetted datasets.

| **Replicate** | **model** | **Number of generations (mil)** | **Tree basal** |
| --- | --- | --- | --- |
| 1 | GTR+G | 4 | Australia & Keurbooms |
| 2 | GTR+G | 2,875 | Egypt |
| 3 | GTR+I | 3,435 | Australia & Keurbooms |
| 4 | GTR+G | 6 | Australia |
| 5 | GTR+G | 6 | Australia & Keurbooms |
| 6 | GTR+G | 3 | Australia & Keurbooms |
| 7 | GTR+G | 3 | Australia & Keurbooms |
| 8 | GTR+G | 6 | No pattern |
| 9 | GTR+G | 5 | Australia |
| 10 | GTR+G | 3 | Australia & Keurbooms |
| 11 | GTR+G | 6 | Australia & Keurbooms |
| 12 | GTR+G | 5,48 | Keurbooms |
| 13 | GTR+I | 5 | Australia & Keurbooms |
| 14 | GTR+G | 5 | Australia & Keurbooms |
| 15 | GTR+I | 5 | Australia & Keurbooms |
| 16 | GTR+I | 5 | Australia & Keurbooms |
| 17 | GTR+G | 3 | Australia & Keurbooms |
| 18 | GTR+I | 5 | Australia & Keurbooms |
| 19 | GTR+G | 3 | Australia & Keurbooms |
| 20 | GTR+G | 5 | Egypt |

Table S3: Pairwise genetic distances of the mt*COI* gene of *Diopatra aciculata* used in this study.

|  | *D. aciculata* Knysna | *D. aciculata* Keurbooms | *D. aciculata* Swartkops | *D. aciculata* Australia | *D. aciculata* Egypt | *Lysidice natalensis* |
| --- | --- | --- | --- | --- | --- | --- |
| *D. aciculata* Knysna |  |  |  |  |  |  |
| *D. aciculata* Keurbooms | 0.006 |  |  |  |  |  |
| *D. aciculata* Swartkops | 0.005 | 0.005 |  |  |  |  |
| *D. aciculata* Australia | 0.017 | 0.016 | 0.015 |  |  |  |
| *D. aciculata* Egypt (Genbank) | 0.015 | 0.015 | 0.014 | 0.026 |  |  |
| *Lysidice natalensis* (Outgroup) | 0.458 | 0.459 | 0.455 | 0.489 | 0.470 |  |

Figure S1: A Bayesian 50% majority-rule consensus tree based on the cytochrome oxidase subunit one (COI) gene of the *D. aciculata* (Figure 2: Clade A) collected in South Africa (Knysna, Keurbooms, and Swartkops Estuaries), Egypt and Australia. Support for clades: posterior probabilities (Bayesian likelihood).

**Figure S2:** The Bayesian 50% majority-rule consensus trees of 20 randomly subsetted sequences based on the cytochrome oxidase subunit one (COI) gene of the *D. aciculata* collected in South Africa (n = 30; Knysna, Keurbooms, and Swartkops Estuaries), Egypt (n = 4) and Australia (n = 10). Support for clades: posterior probabilities (Bayesian likelihood). Number 10 was used for further analysis.
